# The National Park

**Published:** 1883-06

**Authors:** 


### THE NATIONAL PARK.

In the northwestern corner of the Territory of Wyoming, bordering on Montana and Idaho, lies a tract of country about fifty-five by sixty-five miles in extent, possessing a greater combination of remarkable features than any other known area of like dimensions under the sun. It contains 3,578 square miles. Its elevation above the sea level is from six thousand to fourteen thousand feet. It lies mainly, but not entirely, on the east side of the main range of the Rocky Mountains. By act of Congress, approved March 1, 1872, this tract was withdrawn forever from sale and set apart as a permanent pleasure-ground for the amusement and instruction of the people, under the designation of the Yellowstone National Park. The grandeur and variety of its scenery, the salubrity of its summer climate, and the health-giving qualities or its thermal waters will, within a few years, make it the Mecca of the tourist, pleasure-seeker, and invalid from all parts of the civilized world. Among its innumerable attractions are some of the grandest cataracts, cascades, cañons and mountain summits on the continent. Its spoutings geysers in number and magnitude exceed all others known. Its numerous mud springs, solfataras, fumeroles, and beautifully terraced hot springs are beyond description in the magnitude and splendor of their decoration and action.

Our purpose in referring to the park was not so much to attempt a description of its really indescribable wonders as to call attention to the work of vandalism already inaugurated within it by tourists and visitors. Many of the magnificent structures built up by the action of the hot springs and geysers are being disfigured and destroyed by trophy-hunters and others, actuated too often, it is to be feared, by a pure love of destruction. This shameless raid upon the varied glories of the "Wonderland" should at once be stopped by the strong arm of the law. Congress should promptly take such action as will protect and preserve the decorations that Nature for ages past has treasured up among these "everlasting hills," and in the radiant valleys of the upper Yellowstone. A resolution was passed at the recent meeting of the American Association for the Advancement of Science, calling upon our national authorities to act in this matter. It is a subject of quite as much interest to educators as to men of scienee, inasmuch as the park may be justly regarded as a vast museum whose unlimited resources are capable of illustrating almost every object of thought or subject of study within the range of created existences. Let our educators and friends of education, therefore, add their voices and votes to those of the scientists in the effort to preserve from desecration, and for the high purposes of instruction, the grandest heritage of natural sublimity, beauty and utility ever bestowed upon man.—*The Educational Weekly.*

---

Growing Beautiful.—Persons may outgrow disease and become healthy by proper attention to the laws of their physical constitution. By moderate and daily exercise men may become active and strong in limb and muscle. But to grow beautiful, how? Age dims the lustre of the eye, and pales the roses on beauty's cheek ; while crowfeet, and furrows, and wrinkles, and lost teeth, and gray hairs, and bald head, and tottering limbs, and limping most sadly mar the human form divine. But dim as the eye is, as pallid and sombre as may be the face of beauty, and frail and feeble that once strong, erect, and manly body, the immortal soul, just fledging its wings for its home in heaven, may look out through those faded windows as beautiful as the dew-drop of a Summer's morning, as melting as the tears that glisten in affection's eye, by growing kindly, by cultivating sympathy with all human kind, by cherishing forbearance towards the follies and foibles of our race, and feeding, day by day, on that love to God and man which lifts us from the earth and makes us akin to angels.

